# Growth and Accumulation of Secondary Metabolites in Perilla as Affected by Photosynthetic Photon Flux Density and Electrical Conductivity of the Nutrient Solution

**DOI:** 10.3389/fpls.2017.00708

**Published:** 2017-05-04

**Authors:** Na Lu, Emmanuel L. Bernardo, Chayanit Tippayadarapanich, Michiko Takagaki, Natsuko Kagawa, Wataru Yamori

**Affiliations:** ^1^Center for Environment, Health and Field Sciences, Chiba UniversityKashiwa, Japan; ^2^Masikhay MicroPlants NurseryLos Baños, Philippines; ^3^Department of Horticulture, Faculty of Agriculture, Kasetsart UniversityBangkok, Thailand; ^4^Department of Biological Sciences, Graduate School of Science, University of TokyoTokyo, Japan

**Keywords:** perilla, PPFD, EC, secondary metabolites, PUE

## Abstract

The global demand for medicinal plants is increasing. The quality of plants grown outdoors, however, is difficult to control. Myriad environmental factors influence plant growth and directly impact biosynthetic pathways, thus affecting the secondary metabolism of bioactive compounds. Plant factories use artificial lighting to increase the quality of medicinal plants and stabilize production. Photosynthetic photon flux density (PPFD) and electrical conductivity (EC) of nutrient solutions are two important factors that substantially influence perilla (*Perilla frutescens*, Labiatae) plant growth and quality. To identify suitable levels of PPFD and EC for perilla plants grown in a plant factory, the growth, photosynthesis, and accumulation of secondary metabolites in red and green perilla plants were measured at PPFD values of 100, 200, and 300 μmol m^-2^ s^-1^ in nutrient solutions with EC values of 1.0, 2.0, and 3.0 dS m^-1^. The results showed significant interactive effects between PPFD and EC for both the fresh and dry weights of green perilla, but not for red perilla. The fresh and dry weights of shoots and leafy areas were affected more by EC than by PPFD in green perilla, whereas they were affected more by PPFD than by EC in red perilla. Leaf net photosynthetic rates were increased as PPFD increased in both perilla varieties, regardless of EC. The perillaldehyde concentration (mg g^-1^) in red perilla was unaffected by the treatments, but accumulation in plants (mg per plant) was significantly enhanced as the weight of dry leaves increased. Perillaldehyde concentrations in green perilla showed significant differences between combinations of the highest PPFD with the highest EC and the lowest PPFD with the lowest EC. Rosmarinic acid concentration (mg g^-1^) was increased in a combination of low EC and high PPFD conditions. Optimal cultivation conditions of red and green perilla in plant factory will be discussed in terms of plant growth and contents of medicinal ingredients.

## Introduction

The global demand for medicinal plants such as perilla is increasing (Uniyal et al., 2000; Schippmann et al., 2002; Bodeker and Burford, 2007; Payyappallimana, 2010). Perilla (*Perilla frutescens*, Labiatae) is a popular species that has both culinary and medicinal utility (Brenner, 1993; Yu, 1997). Secondary metabolites in perilla plants, such as perillaldehyde and rosmarinic acid, reportedly have the potential to prevent disease, particularly due to their anti-allergic, anti-inflammatory, and antidepressant effects (Makino et al., 2003; Sanbongi et al., 2004; Zhang et al., 2008; Ito et al., 2011; Igarashi and Miyazaki, 2013). Perillaldehyde is a major compound in the essential oil extracted from perilla plants (Ito et al., 1999). As the unique flavor of perilla originates from perillaldehyde and other monoterpenes, the concentration of perillaldehyde in perilla leaves is important as a food-flavoring culinary herb. In addition, the concentration of perillaldehyde is pharmaceutically important for antibacterial activity, as noted in the Japanese Pharmacopeia (Pharmaceutical and Medical Device Regulatory Science Society of Japan [PMRJ], 2016). Rosmarinic acid is commonly found in Labiatae family plants and possesses various phenolic bioactivities that include anti-Alzheimer’s disease, antiviral, antibacterial, anti-inflammatory, and antioxidant qualities (Petersen and Simmonds, 2003; Hamaguchi et al., 2009; Ono et al., 2012). Perilla extracts enriched with rosmarinic acid exert a beneficial anti-allergic effect (Takano et al., 2004). Thus, the concentrations of perillaldehyde and rosmarinic acid in perilla are important for their culinary and clinical applications.

Perilla plants are mainly obtained from outdoor cultivation sites or wild areas. However, the quality of plants grown outdoors is very difficult to control, so the concentrations of the bioactive compounds may vary widely with cultivation conditions, such as location, season (Natsume et al., 2006; Iwai et al., 2010), cultivar (e.g., green or red perilla) (Martinetti et al., 2012), and plant part (Chauhan et al., 2013). Among the targeted secondary metabolites produced in perilla, perillaldehyde and rosmarinic acid have different biosynthetic pathways and distinctive functional roles in the plant. The accumulation of these metabolites may be increased independently, and rather unpredictably, as a response to individual or combinations of abiotic environmental stresses. Thus, a sustainable medicinal plant production system that can manage environmental factors is needed to obtain a stable supply of perilla plants with uniform quality.

Environmental factors such as temperature, humidity, light, and the supply of water, minerals and CO_2_ influence plant growth and have a direct impact on the biochemical pathways that affect the metabolism of secondary products (Akula and Ravishankar, 2011). The ability to control environmental factors using plant factory technology has allowed producers to maintain complete control of their production systems. A plant factory is a plant production facility that provides growers the advantage of controlling environmental factors that control plant growth and development (Yamori et al., 2014; Kozai et al., 2015). In many countries, such as Japan, USA, China, and Korea, plant factories provide year-round high quantity and quality commercial production of leafy vegetables, herbs, and transplants. With plant factory technology, the levels of pharmacological and economic interest in plant production of phytochemicals can attain the desired level while maintaining strict quality standards established for the food and drug industries, which can elevate the mission of plant factories from one of producing plants solely for food, to also producing plants as medicine.

One disadvantage of a plant factory is the high setup and operation cost, particularly the cost of lighting. The cost for the electrical energy used by artificial lighting in plant factories can exceed 25% of total production costs (Kozai, 2013). Light is one of the most important factors affecting plant growth and quality. Identifying the optimal light intensity level that promotes growth is critical for operating a plant factory at low cost. Thus, the effects of light quality and intensity have been the subjects of much research. Nishimura et al. (2007) found that under red light conditions, higher light intensity enhanced the growth of *Hypericum perforatum* L., but reduced the content of both hypericin and pseudohypericin in the plant tissue. The additional UV–B suppressed the growth and anthocyanin production in red perilla leaves (Nishimura et al., 2008). Another study showed that green light enhanced the concentrations of perillaldehyde and limonene in perilla leaf tissues, but suppressed plant growth overall, which resulted in lower net contents of perillaldehyde and limonene per plant compared with plants grown under a mix of blue and red light (Nishimura et al., 2009). Exposure to a long photoperiod of 16 h compared with 12 h enhanced the rosmarinic acid accumulation in peppermint and spearmint plants (Fletcher et al., 2010). Enhanced accumulation of rosmarinic acid was reported in red perilla cultivated under artificial light comprising red laser diode and blue LED followed by UV–A irradiation (Iwai et al., 2010).

Nutrient uptake is generally affected by photosynthetic photon flux density (PPFD) (Poorter and Nagel, 2000; Vendrame et al., 2004), so a plant’s nutrient requirements should also be investigated when different PPFDs are considered. Total values for phenolic content and rosmarinic acid content per shoot weight in fresh oregano (*Origanum vulgare* L.) were increased by nutrient deficiency (Lattanzio et al., 2009). The wide array of literature dedicated to light and nutrition management reflects the value of these environmental factors in plant cultivation (Poorter and Nagel, 2000; Cronin and Lodge, 2003; Gent, 2003; Vendrame et al., 2004; Wu and Kubota, 2008; Nishimura et al., 2009). However, the majority of studies on hydroponically grown medicinal plants have tended to focus on only one environmental factor, and studies concerned with optimizing the nutrient content have tended to direct attention toward the effect of increasing the electrical conductivity (EC) of the nutrient solution. The prescribed EC for optimal availability of essential nutrients in solution ranges between 1.5 and 3.5 dS m^-1^, but this may vary according to the crop species and phenological stage (Resh, 2015). A study by Simeunovic (2002) that tested 1.6, 2.1, and 2.5 dS m^-1^ showed that nutrient availability influenced resource partitioning but did not influence the leaves and flowers of *Achillea millefolium* and *Tanacetum parthenium*. In the same study, an intermediate EC of 2.1 dS m^-1^ caused *Leonurus sibiricus* to allocate more biomass to its vegetative portions. In hydroponically grown basil, the levels of rosmarinic acid varied according to the variety as well as to the highest concentration of rosmarinic acid found during flowering (Kiferle et al., 2011). The salinity of substrates as it relates to crop growth and yield is also a subject of interest (Grattan and Grieve, 1998; Savvas and Lenz, 2000; Koyro, 2006), but only a limited number of studies have used medicinal plants as test crops. No study has reported the effect that combinations of PPFD and EC might exert on perilla. The objective of this study was to investigate the effects of PPFD and EC on the growth and accumulation of secondary metabolites in green and red perilla plants and to determine the optimal combinations of PPFD and EC to achieve the highest production and accumulation of metabolites. Photosynthetically active radiation (PAR) use efficiency (PUE) is also discussed in terms of dry plant mass.

## Materials and Methods

### Plant Material

Green perilla (*P. frutescens* var. *crispa* f. *viridis* Makino; Takii Seed Co., Ltd, Kyoto, Japan) and red perilla (*P. frutescens* (L.) Britton. var. *acuta* Kudo f. *crispa* Makino; 0657–79TS, National Institute of Biomedical Innovation, Osaka, Japan) seeds were sown in rockwool cubes (125 cm^3^) in a cultivation room. PPFD was set to 150 μmol m^-2^ s^-1^ with a photoperiod of 16 h per day provided by cool white fluorescent lamps (FHF32 EX-N-H, Panasonic, Co., Ltd, Japan), and the plants were irrigated with a nutrient solution (Otsuka hydroponic composition, Otsuka Chemical Co. Ltd, Osaka, Japan) (Otsuka formula in **Table 1**). The EC and pH of the nutrient solution were adjusted to 1.2 dS m^-1^ and 6.0, respectively. Air temperature, relative humidity, and CO_2_ concentration were set to 25/20°C (light/dark periods), 60–80%, and 400 μmol mol^-1^, respectively.

**Table 1:** Otsuka Formula.

Composition	Content (%)	Composition	Content (%)
N	21	Fe	0.18
P_2_O_5_	8	Cu	0.002
K_2_O	27	Zn	0.006
MgO	4	Mo	0.002
CaO	23	MnO	0.1
		B_2_O_3_	0.1

### Treatments

Three weeks after sowing, red perilla and green perilla seedlings were transplanted into a walk-in type plant factory (2.9 m × 2.0 m × 2.3 m in LWH) and subjected to three PPFD levels (100, 200, and 300 μmol m^-2^ s^-1^) with a photoperiod of 16 h per day supplied by cool white fluorescent lamps and three EC levels (1.0, 2.0, and 3.0 dS m^-1^, Otsuka formula as above, **Table 1**) for 5 weeks. The PPFD was measured at the surface of the rockwool cubes using a light meter (LI 250A, LI-190R; Li-Cor Inc., Lincoln, NE, USA) before placing the plants. The experiment was set up in a 3 × 3 full factorial in split plot design with PPFD as the main plot and EC levels as subplot, and each treatment contained 18 plants. Three nutrient solution tanks (EC of 1.0, 2.0, and 3.0 dS m^-1^ for each) were prepared beside the cultivation system. The plants were irrigated every 2 days from the bottom using fresh nutrient solution from each tank. The overflowed nutrient solution was discarded after each rockwool cube was saturated. Air temperature, relative humidity, and CO_2_ concentration were set at 23/20°C (light/dark periods), 60–80%, and 1000 μmol mol^-1^, respectively.

### Measurements

#### Growth Parameters

The plants were harvested 5 weeks after transplant. Leaf and stem fresh weights, and total leaf area were determined. The leaf and stem samples were placed in an 80°C oven for 1 week to determine dry weight. Leaf area was determined using a Li–3000 leaf area meter (Li-Cor, Lincoln, NE, USA). Leaf mass per area (LMA) was determined as leaf dry weight divided by leaf area. PAR use efficiency (PUE) (g mol^-1^) was calculated by dividing plant shoot dry weight by the integrated PAR received by the plant during the cultivation period after transplant.

#### Gas-Exchange Parameters

Simultaneous gas exchange and chlorophyll fluorescence were measured at the growth light intensity with an open gas exchange system (LI-6400, Li-Cor, Inc., Lincoln, NE, USA) and the integrated fluorescence chamber head (LI-6400-40, Li-Cor, Inc., Lincoln, NE, USA) (Yamori et al., 2011). The electron transport rate (ETR) from chlorophyll a fluorescence was calculated as ETR = 0.5 × absI × ΦPSII, where 0.5 is the fraction of absorbed light reaching photosystem II, absI is absorbed irradiance taken as 0.85 of incident irradiance and ΦPSII is the quantum yield of photosystem II [ΦPSII = (F_m_′-*F*′)/F_m_′]. Ten plants were selected randomly from each treatment 4 weeks after transplant, and the youngest fully expanded leaves from each treatment were used for measurements. Leaf temperature, relative humidity, and the CO_2_ concentration were set to 23°C, 65%, and 1000 μmol mol^-1^, respectively.

#### Perillaldehyde, Rosmarinic Acid, and Anthocyanin Contents

Analysis of perillaldehyde and rosmarinic acid of perilla leaves was conducted according to Japanese Pharmacopeia (Pharmaceutical and Medical Device Regulatory Science Society of Japan [PMRJ], 2016) and methods described in literature (Natsume et al., 2006; Jirovsk y et al., 2007; Nishimura et al., 2009; Öztürk et al., 2010; Sevindik et al., 2015) with modifications. The leaves used for gas-exchange measurement were sampled and dried at 30°C for 2 weeks. The dried perilla leaves were ground to powder and filtered through a sieve. A 10.00–10.50 mg sample was weighed accurately and transferred to a 1.5 mL tube. Methanol (1 mL) was added, mixed for 10 min at 2,000 rpm and 15°C using an Eppendorf ThermoMixer (Hamburg, Germany), and centrifuged for 5 min. To the residue, methanol (1 mL × 2) was added, and the same extract manner was performed twice. The extracts (about 3 mL) were combined and transferred to a 5 mL volumetric flask and diluted with methanol to a 5 mL total volume. The solution was filtered through Agilent 0.2 μm nylon syringe filters (Agilent Technologies Inc., Palo Alto, CA, USA) to prepare the samples for high performance liquid chromatography (HPLC). The HPLC conditions for perillaldehyde were TSKgel ODS-80T_M_ column (5 μm, 4.6 mm × 150 mm) (TOSOH, Tokyo, Japan); temperature, 40°C; flow rate, 1.0 mL/min; run time, 30 min; detector wavelength, 230 nm; mobile phase, 40% acetonitrile; and injection volume, 10 μL. The HPLC conditions for rosmarinic acid were TSKgel ODS-80T_M_ column (5 μm, 4.6 mm × 150 mm) (TOSOH, Tokyo, Japan); temperature, 40°C; flow rate, 1.0 mL/min; run time, 20 min; detector wavelength, 330 nm; mobile phase, 20% acetonitrile/0.1% formic acid; and injection volume, 10 μL. The perillaldehyde and rosmarinic acid standards were dissolved in methanol. Perillaldehyde and rosmarinic acid contents per leaf dry weight (hereafter, perillaldehyde or rosmarinic acid concentration) were estimated by dividing the perillaldehyde and rosmarinic acid contents in samples by sample weight.

The perillaldehyde and rosmarinic acid analytical standard, methanol (LC-MS grade), and water (LC-MS grade) were purchased from Wako Pure Chemical Industries (Osaka, Japan). Acetonitrile (HPLC grade) was obtained from Sigma–Aldrich Japan (Tokyo, Japan). Formic acid was purchased from Kanto Chemical Co., Inc. (Tokyo, Japan). HPLC was performed on a Shimadzu LC-20A Prominence system equipped with a SIL-20AC autosampler and a SPD-20A PDA detector using LabSolutions software (Shimadzu, Kyoto, Japan). The leaf anthocyanin concentration (anthocyanin readings, ACM-200plus, Opti-Sciences, Inc., USA) was determined at harvest for the youngest completely expanded leaves per plant, and the mean of three readings was recorded.

### Statistical Analysis

All experiments were repeated twice with three replications for each treatment. Nine to twelve plants were sampled from each treatment to evaluate overall growth and the photosynthetic parameters, and four to five plants were sampled from each treatment to determine the perillaldehyde and rosmarinic acid concentrations. The data were subjected to analysis of variance and the means were compared between treatments using Tukey’s test in SPSS statistical software (IBM SPSS Statistics, Version 19.0. Armonk, NY, USA: IBM Corp.). A *p*-value < 0.05 was considered significant.

## Results

### Plant Growth

#### Green Perilla

**FIGURE 1:**
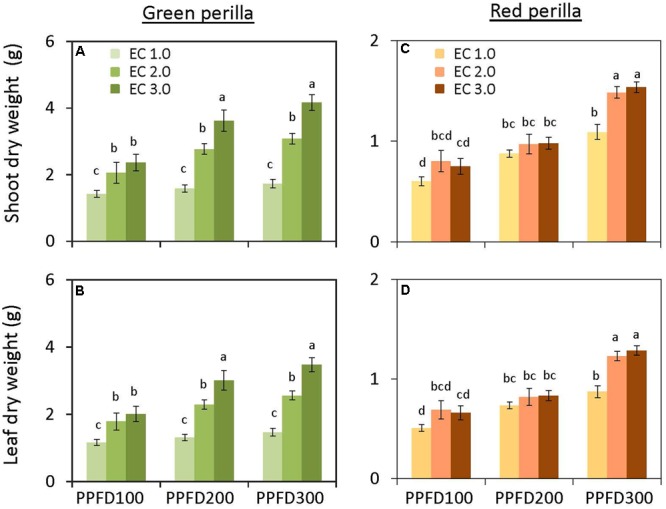
**Shoot dry weight (A) and leaf dry weight **(B)** in green perilla, and shoot dry weight **(C)** and leaf dry weight **(D)** in red perilla plants after 5 weeks of cultivation under different photosynthetic photon flux density (PPFD) and electrical conductivity (EC) treatments.** Values are mean ± standard error (*n* = 10–12). Different letters indicate significant differences between the treatments at *P* < 0.05, as determined by Tukey’s test.

**FIGURE 2:**
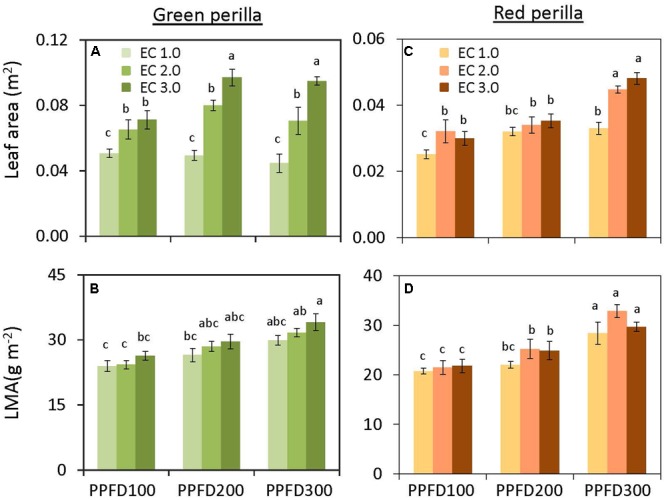
**Total leaf area (A) and leaf mass per area (LMA) **(B)** in green perilla, and total leaf area **(C)** and leaf mass per area **(D)** in red perilla plants after 5 weeks of cultivation under different PPFD and EC treatments.** Values are mean ± standard error (*n* = 10–12). Different letters indicate significant differences between treatments at *P* < 0.05, as determined by Tukey’s test.

Shoot and leaf dry weights were not affected by PPFD at EC values of 1.0 and 2.0 dS m^-1^ (**Figures 1A,B**). However, shoot and leaf dry weights at an EC of 3.0 dS m^-1^ were significantly greater at a PPFD of 200 or 300 μmol m^-2^ s^-1^ than those at a PPFD of 100 μmol m^-2^ s^-1^. Similar trends were observed for shoot and leaf fresh weights (**Supplementary Figures S1A,B**) and leaf area (**Figure 2A**). LMA tended to increase with an increase in PPFD under the same EC, but was not significantly affected by EC under the same PPFD (**Figure 2B**). Leaf-shoot ratio was not affected by PPFD or EC treatments (**Supplementary Figure S2A**). Significant effects of PPFD, EC, and an interaction between the two factors were observed for shoot dry weight (*P* < 0.001, *P* < 0.001, and *P* = 0.0104, respectively), leaf dry weight (*P* < 0.001, *P* < 0.001, and *P* = 0.0139, respectively) (**Figures 1A,B**), shoot fresh weight (*P* < 0.001, *P* < 0.001, and *P* = 0.0057, respectively), and leaf fresh weight (*P* < 0.001, *P* < 0.001, and *P* = 0.0127, respectively) (Supplementary Table S1).

#### Red Perilla

There was no interaction between PPFD and EC on shoot and leaf dry weights (Supplementary Table S1). The individual main effects, however, were significant. Both shoot and leaf dry weights tended to increase with an increase in either PPFD or EC (**Figures 1C,D**). Similar trends were observed for shoot and leaf fresh weights (**Supplementary Figures S1C,D**), leaf area (**Figure 2C**), and LMA (**Figure 2D**). Leaf-shoot ratio was not affected by PPFD or EC treatments (**Supplementary Figure S2B**).

#### Variety Differences

The biomass production and leaf area of green perilla were 58 ∼ 270% and 35 ∼ 175% higher than those of red perilla under the same growth conditions.

### Gas-Exchange Parameters

**FIGURE 3:**
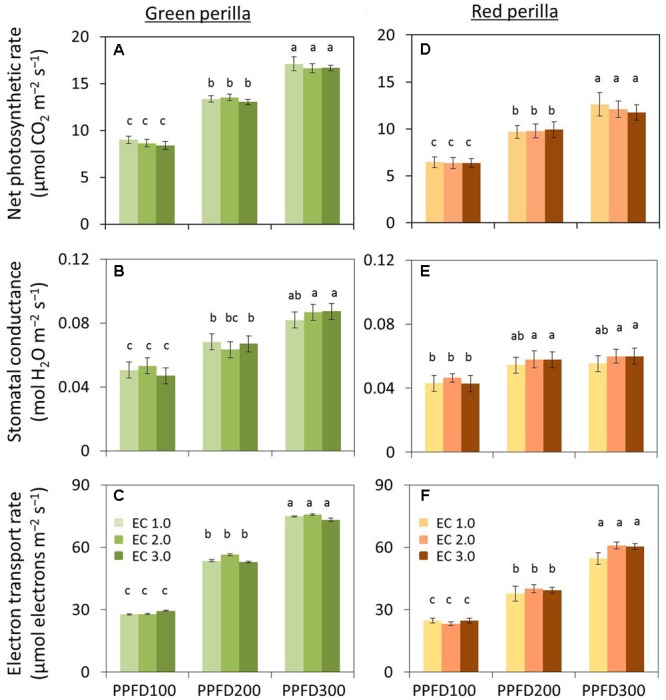
**Effects of different PPFD and EC treatments on net photosynthetic rate (A), stomatal conductance **(B)**, and electron transport rate (ETR) **(C)** in green perilla, and net photosynthetic rate **(D)**, stomatal conductance **(E)**, and ETR **(F)** in red perilla plants 4 weeks after transplant.** PPFD was 100, 200, and 300 μmol m^-2^s^-1^. Leaf temperature and CO_2_ concentration were 23 ± 1°C and 1,000 ± 5 μL L^-1^, respectively. Values are mean ± standard error (*n* = 10–12). Different letters indicate significant differences between treatments at *P* < 0.05, as determined by Tukey’s test.

Net photosynthetic rate, stomatal conductance, and photosynthetic ETR increased with increases in PPFD in green perilla (**Figures 3A–C**) and red perilla (**Figures 3D–F**), regardless of EC.

#### Variety Differences

Net photosynthetic rate, stomatal conductance, and photosynthetic ETR in green perilla were 32 ∼ 42%, 17 ∼ 48%, and 12 ∼ 42%, respectively, higher than those of red perilla under the same growth conditions.

### Perillaldehyde, Rosmarinic Acid, and Anthocyanin Contents

#### Green Perilla

**FIGURE 4:**
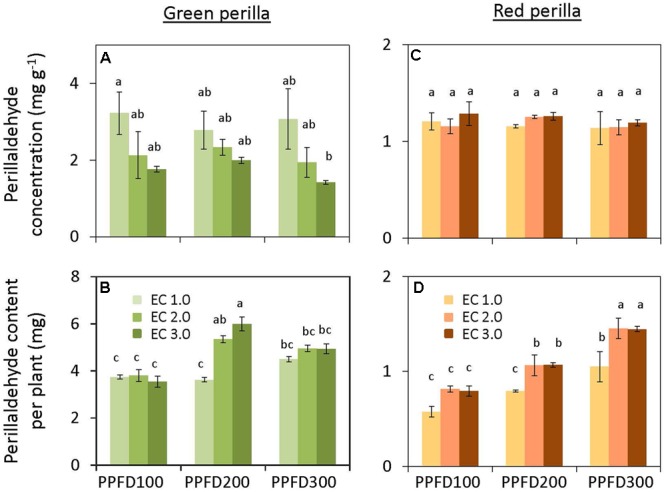
**Perillaldehyde concentration (perillaldehyde content per unit leaf dry weight) (A) and perillaldehyde content per plant **(B)** in green perilla, and perillaldehyde concentration **(C)** and perillaldehyde content per plant **(D)** in red perilla plants after 5 weeks of cultivation under different PPFD and EC treatments.** Values are mean ± standard error (*n* = 4–5). Different letters indicate significant differences between treatments at *P* < 0.05, as determined by Tukey’s test.

**FIGURE 5:**
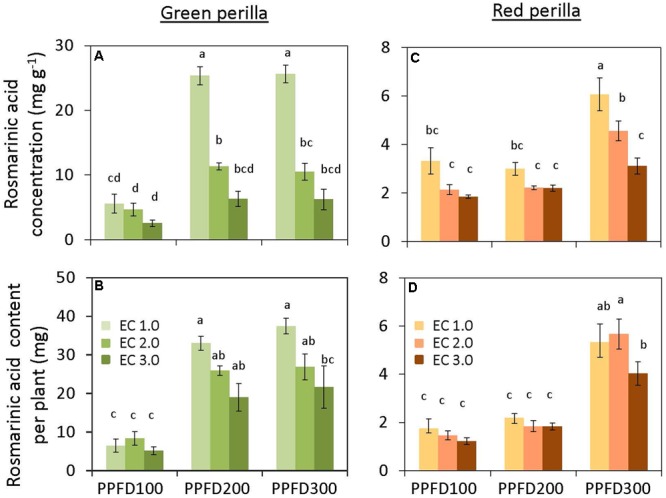
**Rosmarinic acid concentration (rosmarinic acid content per unit leaf dry weight) (A) and rosmarinic acid content per plant **(B)** in green perilla, and rosmarinic acid concentration **(C)** and rosmarinic acid content per plant **(D)** in red perilla plants after 5 weeks of cultivation under different PPFD and EC treatments.** Values are mean ± standard error (*n* = 4–5). Different letters indicate significant differences between treatments at *P* < 0.05, as determined by Tukey’s test.

**FIGURE 6:**
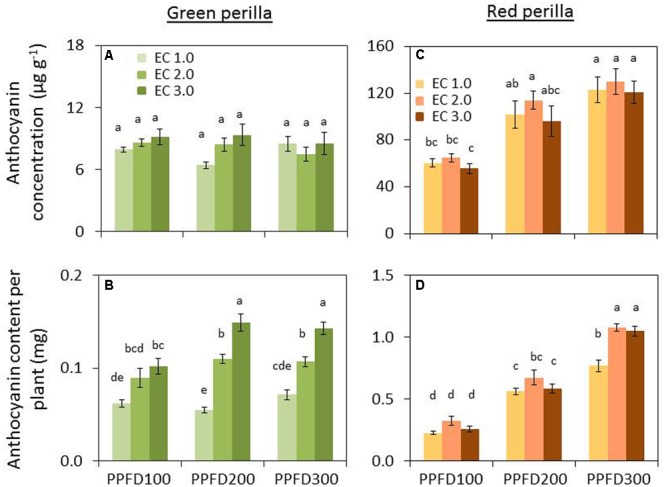
**Anthocyanin concentration (anthocyanin content per unit leaf dry weight) (A) and anthocyanin content per plant **(B)** in green perilla, and anthocyanin concentration **(C)** and anthocyanin content per plant **(D)** in red perilla plants after 5 weeks of cultivation under different PPFD and EC treatments.** Values are mean ± standard error (*n* = 4–5). Different letters indicate significant differences between treatments at *P* < 0.05, as determined by Tukey’s test.

Perillaldehyde concentration was not affected by PPFD under the same EC (**Figure 4A**), but tended to decrease with an increase in EC. Perillaldehyde content per plant was not affected by PPFD under an EC of 1.0 dS m^-1^, however, it was affected by PPFD under EC values of 2.0 and 3.0 dS m^-1^ (**Figure 4B**). Rosmarinic acid concentration increased significantly under PPFD values of 200 and 300 μmol m^-2^ s^-1^ at EC values of 1.0 and 2.0 dS m^-1^, compared with that at a PPFD of 100 μmol m^-2^ s^-1^, whereas rosmarinic acid concentration was not affected by PPFD under an EC of 3.0 dS m^-1^ (**Figure 5A**). Rosmarinic acid content per plant also increased under PPFD values of 200 and 300 μmol m^-2^ s^-1^ in the same EC, compared with that of a PPFD of 100 μmol m^-2^ s^-1^, and higher rosmarinic acid contents were obtained under lower ECs (**Figure 5B**). Significant effects of PPFD, EC, and an interaction between the two factors were observed for rosmarinic acid concentration (*P* < 0.001, *P* < 0.001, and *P* < 0.0001, respectively) (Supplementary Table S2). Anthocyanin concentration was not affected by PPFD or EC treatments in green perilla, but anthocyanin content per plant increased with increases in EC under a PPFD of 200 and 300 μmol m^-2^ s^-1^ (**Figures 6A,B**).

#### Red Perilla

Perillaldehyde concentration was not affected by PPFD or EC (**Figure 4C**). Perillaldehyde content per plant increased with increases in PPFD (**Figure 4D**). Perillaldehyde content per plant was not affected by EC under a PPFD of 100 μmol m^-2^ s^-1^, however, was increased by EC of 2.0 and 3.0 dS m^-1^ compared to EC of 1.0 dS m^-1^ under a PPFD of 200 and 300 μmol m^-2^ s^-1^. Rosmarinic acid concentration increased significantly under a PPFD of 300 μmol m^-2^ s^-1^ at EC values of 1.0 and 2.0 dS m^-1^, but was not affected by PPFD under an EC of 3.0 dS m^-1^ (**Figure 5C**). Rosmarinic acid content per plant was higher at a PPFD of 300 μmol m^-2^ s^-1^ than that at PPFD values of 100 or 200 μmol m^-2^ s^-1^ under all ECs (**Figure 5D**). The highest anthocyanin concentration were obtained at PPFD of 200, and 300 μmol m^-2^ s^-1^, but was not affected by EC in red perilla (**Figure 6C**). Anthocyanin content per plant increased with increases in PPFD regardless of EC (**Figure 6D**). Anthocyanin content per plant was not affected by EC under a PPFD of 100 or 200 μmol m^-2^ s^-1^, however, was increased by EC of 2.0 and 3.0 dS m^-1^ compared to EC of 1.0 dS m^-1^ under a PPFD of 300 μmol m^-2^ s^-1^. No significant interaction effects between PPFD and EC were observed for perillaldehyde concentration, rosmarinic acid concentration, or anthocyanin concentration (Supplementary Table S2).

#### Variety Differences

Perillaldehyde and rosmarinic acid concentrations in leaves of green perilla were 19 ∼ 270% and 37 ∼ 745% higher than those of red perilla; however, red perilla leaves contained more than five times higher anthocyanin concentration than green perilla leaves under the same growth conditions.

### PAR Use Efficiency (PUE)

#### Green Perilla

**FIGURE 7:**
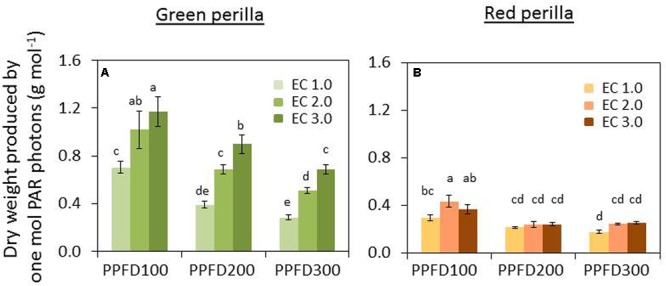
**Effects of different PPFD and EC treatments on photosynthetically active radiation (PAR) use efficacy (PUE) in green perilla (A) and red perilla** **(B)**. PUE (g mol^-1^) was calculated by dividing plant shoot dry weight by the integrated PAR photons received by the plant during the cultivation period after transplant. Values are mean ± SE (*n* = 10–12). Different letters indicate significant differences between treatments at *P* < 0.05, as determined by Tukey’s test.

PUE decreased with increases in PPFD under the same EC. But PUE increased as EC increased under the same PPFD (**Figure 7A**).

#### Red Perilla

PAR use efficiency decreased with the increase of PPFD from 100 to 200 μmol m^-2^ s^-1^, but no further decrease was observed under a PPFD of 300 μmol m^-2^ s^-1^, under all ECs (**Figure 7B**).

#### Variety Differences

PAR use efficiency of green perilla was 58 ∼ 270% higher than that of red perilla under the same growth conditions.

## Discussion

### Growth Response of Perilla to PPFD and EC

A higher PPFD usually results in a higher photosynthetic rate and greater dry weight gain (Faust and Logan, 1998), and plant nutritional requirements are believed to be higher under higher light intensities than under lower light intensities (Joiner et al., 1981; Nelson, 1996). In our study, the growth parameters of green and red perilla were promoted under higher PPFD values when higher EC values were applied (**Figures 1, 2**). However, the sensitivities to PPFD and EC changed with variety; green perilla growth tended to be more sensitive to EC than to PPFD, whereas red perilla growth tended to be more sensitive to PPFD than to EC. Growth of green perilla was limited by EC values of 1.0 and 2.0 dS m^-1^, and no changes with increasing PPFD. Only when EC was increased to 3.0 dS m^-1^, the fresh/dry weights were increased with increasing PPFD, indicating that a higher EC (3.0 dS m^-1^) enhanced the capacity of green perilla to utilize light. EC of 1.0 dS m^-1^ limited red perilla growth when a high PPFD (300 μmol m^-2^ s^-1^) was applied, and EC value between 2.0 and 3.0 dS m^-1^ should be an optimal range under such high PPFD (**Figures 1, 2**).

The production of green perilla plants was more than two times higher than that of red perilla plants when the same environmental condition was applied (**Figure 1** and **Supplementary Figure S1**), demonstrating the biomass productivity of green perilla was higher than that of red perilla. This is supported by previous research which showed that green perilla cultivars generally grow faster than red ones in open fields (Martinetti et al., 2012). The difference in productivity may be due to the difference in photosynthetic capacity between green perilla and red perilla (**Figure 3**), since plant growth largely depends on photosynthesis (Yamori, 2016; Yamori and Shikanai, 2016; Yamori et al., 2016). The higher photosynthetic capacity of green perilla reflected in higher net photosynthetic rate, stomatal conductance, and ETR per unit leaf area, compared to those in red perilla (**Figure 3**). High stomatal conductance could increase CO_2_ supply into plant leaves, thus improve ETR and net photosynthetic rate. Moreover, plant development also differed between the two varieties. As the green perilla plants had larger total leaf area but similar leaf-shoot ratio, both higher net photosynthetic rate per unit leaf area and larger total leaf area would contribute to higher biomass production in green perilla, compared to red perilla (**Figures 2, 3** and **Supplementary Figure S2**).

### Response of Perillaldehyde, Rosmarinic Acid, and Anthocyanin Contents to PPFD and EC

Perillaldehyde and rosmarinic acid concentrations in green perilla were higher than those in red perilla, whereas, anthocyanin concentration in red perilla was higher than that of green perilla (**Figures 4**–**6**), which highlight varietal differences in secondary metabolite synthesis as it relates to PPFD and EC. A study in hydroponically grown basil showed a similar trend where the two green varieties, ‘Genovese’ and ‘Superbo,’ had higher rosmarinic acid contents than the ‘Dark Opal’ cultivar with purple leaves (Kiferle et al., 2011). Perillaldehyde is produced by the monoterpene biosynthetic pathway. As monoterpenes accumulate in glandular trichomes of perilla plants, perillaldehyde concentration may decrease with increasing LMA. The result showed, however, perillaldehyde concentrations (mg g^-1^) were not changed under treatments in which LMAs had increased by PPFD or EC (**Figures 2B,D, 4A,C**). Therefore, the density of perillaldehyde distributed in leaves has been changed by the effects of EC and/or PPFD. Rosmarinic acid is an ester of two phenolic compounds and is synthesized via the phenylpropanoid pathway. Chen et al. (2011) found that low nutrient supply enhances rosmarinic acid content in *P. vulgaris* leaves. So the nutrient solution level had a strong effect on rosmarinic acid concentration. In the present study, perillaldehyde concentrations were not significantly affected by PPFD or EC, whereas, rosmarinic acid concentrations were affected by PPFD, EC or an interaction between the two factors. The highest concentrations were obtained under the lowest EC (1.0 dS m^-1^) when perilla were grown under the highest PPFD (300 μmol m^-2^ s^-1^) (**Figures 5A,C**). This means that production of rosmarinic acid has been induced in perilla plants by the treatment under EC of 1.0 dS m^-1^ with a PPFD of 300 μmol m^-2^ s^-1^ to combat low nutrient uptake stress.

The perillaldehyde, rosmarinic acid, and anthocyanin contents per plant were related to the effects of the treatments on plant biomass. The present study showed that high perillaldehyde and anthocyanin contents per plant were maintained when plant growth was promoted, whereas rosmarinic acid content per plant was mainly regulated by EC levels. The results obtained in this research demonstrated the possibility of a plant factory to produce perilla plants with both high production and good quality by controlling environmental conditions. To obtain high production in both of plant growth and accumulation of secondary metabolites, the regulation of PPFD and/or EC can be applied differently at different plant growth stages. For example, Ogawa et al. (2012) found that applying regular nutrient solution to leafy vegetables in the former half of their growth period, followed by a potassium free nutrient solution used in the latter half of their growth period significantly reduced the potassium content of plants without influencing plant growth. This principle can be also applicable for medicinal plants production in plant factory. In the early stage of plant growth period, the higher PPFD and EC which could enhance plant growth should be applied to achieve a maximum plant biomass. And a different PPFD or EC, such as high PPFD and low EC, would be used in the latter stage of plant growth period to maximize accumulation of secondary metabolites (such as rosmarinic acid). The results obtained in this research provided substantive evidence and may serve as important references for PPFD and EC applications in perilla plant production in a plant factory.

### The PUE Response to PPFD and EC

The PUE of dry mass varied under the different PPFDs, ECs, and varieties (**Figure 7**) in this research. Wheeler et al. (2008) reported that radiation use efficiencies for several crops were in the range of 0.43–0.64 g mol^-1^. Goto (2009) compared PUEs of four different crops, and found those did not differ significantly among crops. In the present study, the PUE values for total dry mass (roots not included) were 0.31–1.54 g mol^-1^ for green perilla and 0.18–0.44 g mol^-1^ for red perilla. And it was possible to increase PUE by adjusting ECs or PPFDs in perillas. The results in green perilla show that PUE increased significantly under a higher EC (**Figure 7A**). This finding is important for commercial production of medicinal plants and also vegetables in a plant factory. Because the cost in lighting is a large part of total production costs, and EC regulation is relatively lower cost than lighting regulation. It is essential to determine the corresponding EC which can maximize PUE when applying a particular PPFD.

## Conclusion

This study investigated the effects of PPFD and EC on the growth and the accumulation of secondary metabolites in green and red perilla plants. The results show that under the same cultivation condition, plant weights and accumulation of secondary metabolites in green perilla both were higher than that in red perilla. There were significant interactive effects between PPFD and EC for the fresh and dry weights of green perilla, but not for red perilla. Plant growth and development in green perilla were affected more by EC than by PPFD, whereas those in red perilla were affected more by PPFD than by EC. Leaf net photosynthetic rates, stomatal conductances, and photosynthetic ETRs were increased as PPFD increased in both perilla varieties, regardless of EC. Perillaldehyde concentration was not affected by EC or PPFD in red perilla, therefore net perillaldehyde content per plant was increased under high PPFD with high EC when plant biomass was promoted. However, in green perilla, perillaldehyde concentration tended to decreased with an increase in EC, and was accumulated the most under the lowest PPFD with lowest EC and the least under the highest PPFD with highest EC condition. Rosmarinic acid concentration was increased by a combination of a low EC and a high PPFD in both varieties. Anthocyanin content in red perilla was significantly higher than that in green perilla. PUE was increased by increasing the level of EC in green perilla. The results obtained in this research could be an important reference for producers when selecting varieties and applying strategies of PPFD and EC to achieve their goals in terms of biomass production and the accumulation of secondary metabolites.

## Author Contributions

NL and WY conceived and designed the experiments. NL, EB, CT, and NK performed the experiments. NL, EB, CT, NK, and WY analyzed the data and prepared figures and graphs. MT and NK contributed reagents, materials, and analysis tools. NL, NK and WY prepared the manuscript, and all the members contributed extensively to its finalization.

## Conflict of Interest Statement

The authorsdeclare that the research was conducted in the absence of any commercial or financial relationships that could be construed as a potential conflict of interest.

## Supplementary Material

FIGURE S1 **Shoot fresh weight (A)** and leaf fresh weight **(B)** in green perilla, and shoot fresh weight **(C)** and leaf fresh weight **(D)** in red perilla plants after 5 weeks of cultivation under different photosynthetic photon flux density (PPFD) and electrical conductivity (EC) treatments. Values are mean ± standard error (*n* = 10–12). Different letters indicate significant differences between treatments at *P* < 0.05, as determined by Tukey’s test.

FIGURE S2 **Leaf-shoot ratio in green perilla (A)** and red perilla **(B)** plants after 5 weeks of cultivation under different photosynthetic photon flux density (PPFD and EC treatments. Values are mean ± standard error (*n* = 10–12). Different letters indicate significant differences between treatments at *P* < 0.05, as determined by Tukey’s test.
