# Supplementary material for: Growth and Accumulation of Secondary Metabolites in Perilla as Affected by Photosynthetic Photon Flux Density and Electrical Conductivity of the Nutrient Solution
Source: Front Plant Sci. 2017 May 4;8:708. doi: 10.3389/fpls.2017.00708 (PMC5416839; doi:10.3389/fpls.2017.00708)

Supplemental Fig. 1 Shoot and leaf fresh weight

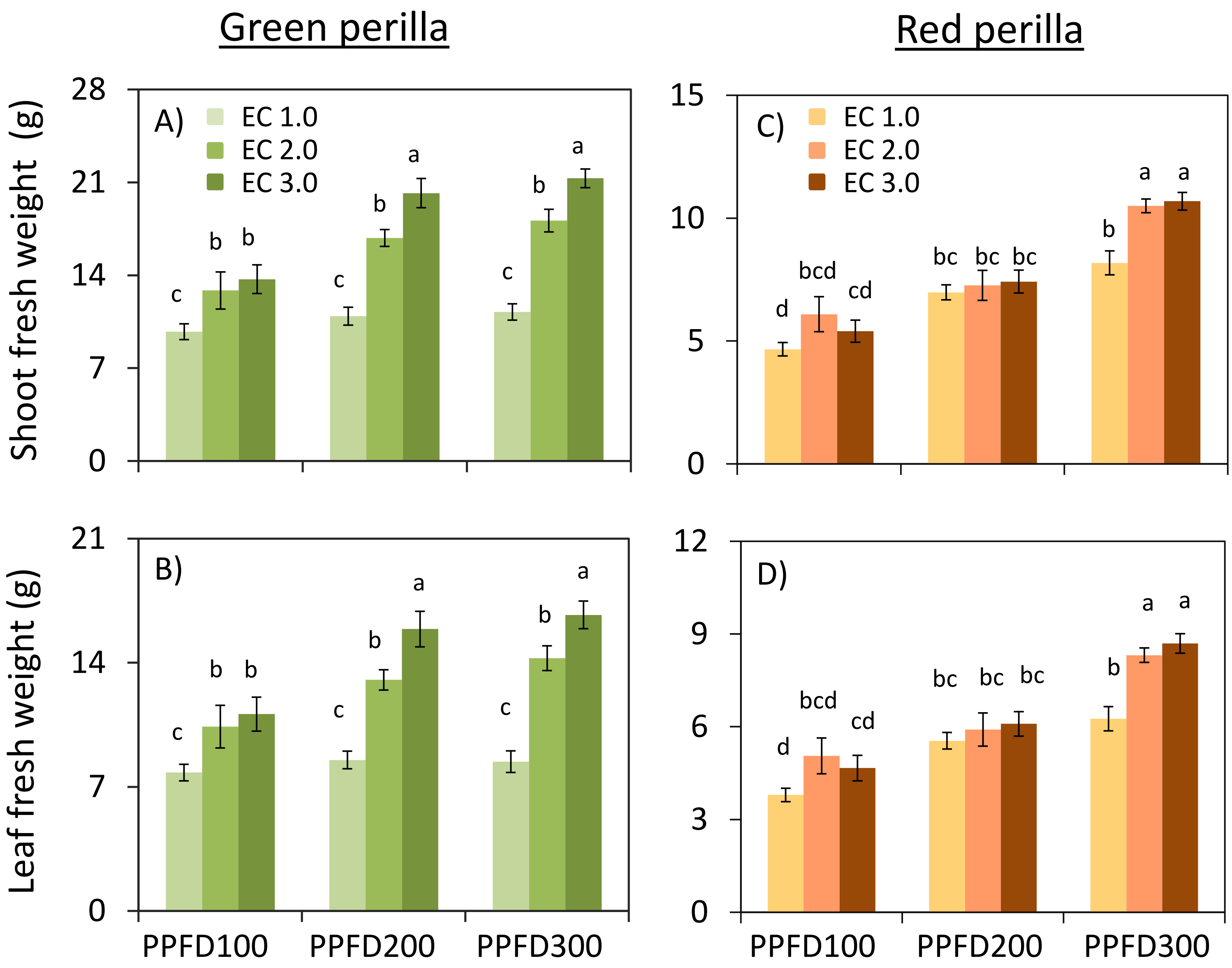

Supplemental Fig. 2

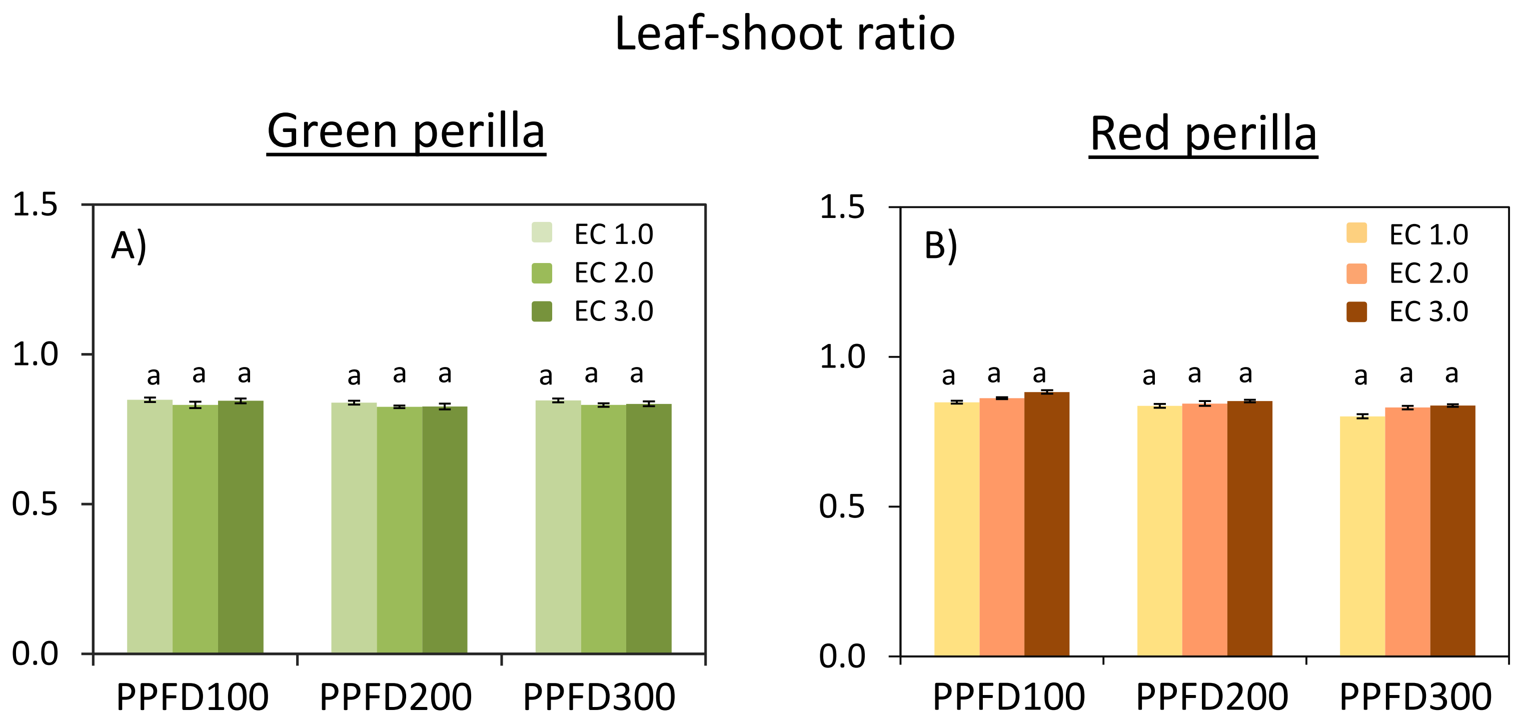

Supplement: FIGURE S1 — Shoot fresh weight (A) and leaf fresh weight (B) in green perilla, and shoot fresh weight (C) and leaf fresh weight (D) in red perilla plants after 5 weeks of cultivation under different photosynthetic photon flux density (PPFD) and electrical conductivity (EC) treatments. Values are mean ± standard error (n = 10–12). Different letters indicate significant differences between treatments at P < 0.05, as determined by Tukey’s test. [file Data_Sheet_2.PDF]
