# Supplementary material for: Growth and Accumulation of Secondary Metabolites in Perilla as Affected by Photosynthetic Photon Flux Density and Electrical Conductivity of the Nutrient Solution
Source: Front Plant Sci. 2017 May 4;8:708. doi: 10.3389/fpls.2017.00708 (PMC5416839; doi:10.3389/fpls.2017.00708)
Supplement: Supplementary file 2 [file Data_Sheet_1.DOCX]

**Supplemental Tables**

**Supplemental table 1.** Analysis of variance of green perilla and red perilla plant growth parameters (shoot dry weight, leaf dry weighit, shoot fresh weight, leaf fresh weight, leaf area, and LMA), photosynthesis parameters (net photosynthetic rate, stomatal conductance, and electron transport rate), and PPDF use efficiency. Plants were subjected to 3 EC levels (1.0, 2.0, and 3.0 dS m^−1^) and grown in 3 PPFDs (100, 200 and 300 µmol m^−2^ s^−1^).

|  |  |  | P value | | | | | | | | | |
| --- | --- | --- | --- | --- | --- | --- | --- | --- | --- | --- | --- | --- |
|  | Source | df | S DW | LDW | S FW | LFW | LA | LMA | Pn | Cond | ETR | PUE |
| Green perilla | Corrected Model | 8 | 0.0000 | 0.0000 | 0.0000 | 0.0000 | 0.0000 | 0.0000 | 0.0000 | 0.0056 | 0.0000 | 0.0000 |
|  | Intercept | 1 | 0.0000 | 0.0000 | 0.0000 | 0.0000 | 0.0000 | 0.0000 | 0.0000 | 0.0000 | 0.0000 | 0.0000 |
|  | PPFD | 2 | 0.0000 | 0.0000 | 0.0000 | 0.0000 | 0.5730 | 0.0000 | 0.0000 | 0.8504 | 0.0000 | 0.0000 |
|  | EC | 2 | 0.0000 | 0.0000 | 0.0000 | 0.0000 | 0.0000 | 0.0036 | 0.0185 | 0.0001 | 0.3651 | 0.0000 |
|  | PPFD x EC | 4 | 0.0104 | 0.0139 | 0.0057 | 0.0127 | 0.3826 | 0.2044 | 0.6512 | 0.8922 | 0.4418 | 0.0591 |
|  |  |  |  |  |  |  |  |  |  |  |  |  |
| Red  perilla | Corrected Model | 8 | 0.0000 | 0.0000 | 0.0000 | 0.0000 | 0.0000 | 0.0000 | 0.0000 | 0.1727 | 0.0000 | 0.0000 |
|  | Intercept | 1 | 0.0000 | 0.0000 | 0.0000 | 0.0000 | 0.0000 | 0.0000 | 0.0000 | 0.0000 | 0.0000 | 0.0000 |
|  | PPFD | 2 | 0.0000 | 0.0000 | 0.0000 | 0.0000 | 0.0000 | 0.0000 | 0.0000 | 0.0091 | 0.0000 | 0.0000 |
|  | EC | 2 | 0.0003 | 0.0001 | 0.0015 | 0.0003 | 0.0001 | 0.0732 | 0.5627 | 0.6458 | 0.9717 | 0.0002 |
|  | PPFD x EC | 4 | 0.1149 | 0.0955 | 0.1167 | 0.1055 | 0.0359 | 0.6410 | 0.7672 | 0.9313 | 0.5858 | 0.0777 |

**Supplemental table 2.** Analysis of variance of green perilla and red perilla plant secondary metabolites (perillaldehyde, rosmarinic acid, anthocyanin concentrations, and those contents per plant). Plants were subjected to 3 EC levels (1.0, 2.0, and 3.0 dS m^−1^) and grown in 3 PPFDs (100, 200 and 300 µmol m^−2^ s^−1^).

|  |  |  | P value | | | | | |
| --- | --- | --- | --- | --- | --- | --- | --- | --- |
|  | Source | df | PA^x^ | RA^y^ | Antho. ^z^ | PA/plant | RA/plant | Antho./plant |
| Green perilla | Corrected Model | 8 | 0.0326 | 0.0000 | 0.1512 | 0.0000 | 0.0000 | 0.0000 |
|  | Intercept | 1 | 0.0000 | 0.0000 | 0.0000 | 0.0000 | 0.0000 | 0.0000 |
|  | PPFD | 2 | 0.7065 | 0.0000 | 0.6516 | 0.0003 | 0.0000 | 0.0001 |
|  | EC | 2 | 0.0010 | 0.0000 | 0.0586 | 0.0000 | 0.0011 | 0.0000 |
|  | PPFD x EC | 4 | 0.7974 | 0.0000 | 0.2253 | 0.0040 | 0.0733 | 0.0031 |
|  |  |  |  |  |  |  |  |  |
| Red  perilla | Corrected Model | 8 | 0.9068 | 0.0000 | 0.0000 | 0.0000 | 0.0000 | 0.0000 |
|  | Intercept | 1 | 0.0000 | 0.0000 | 0.0000 | 0.0000 | 0.0000 | 0.0000 |
|  | PPFD | 2 | 0.6075 | 0.0000 | 0.0000 | 0.0000 | 0.0000 | 0.0000 |
|  | EC | 2 | 0.5051 | 0.0000 | 0.2582 | 0.0000 | 0.0764 | 0.0000 |
|  | PPFD x EC | 4 | 0.9249 | 0.0627 | 0.9823 | 0.7943 | 0.3460 | 0.0061 |

^x^ PA=Perillaldeyde concentration

^y^ RA= Rosmarinic acid concentration

^z^ Antho.= Anthocyanin concentration
